# Analyzing pathogenic (double-stranded (ds) DNA-specific) plasma cells via immunofluorescence microscopy

**DOI:** 10.1186/s13075-015-0811-2

**Published:** 2015-10-21

**Authors:** Oliver Winter, Stephanie Musiol, Melissa Schablowsky, Qingyu Cheng, Laleh Khodadadi, Falk Hiepe

**Affiliations:** Department of Rheumatology and Clinical Immunology, Charité - Universitätsmedizin Berlin, Charitéplatz 1, 10117 Berlin, Germany; Department of Autoimmunology, Deutsches Rheuma-Forschungszentrum, Charitéplatz 1, 10117 Berlin, Germany; Department of Neonatology, Charité - Universitätsmedizin Berlin, Augustenburger Platz 1, 13353 Berlin, Germany

**Keywords:** Autoimmunity, dsDNA, Autoantibodies, Lupus/SLE, Long-lived plasma cells, Histology, Immunofluorescence microscopy

## Abstract

**Introduction:**

While protective plasma cells (PCs) are an important part of the individual’s immune defense, autoreactive plasma cells such as dsDNA-specific plasma cells contribute to the pathogenesis of autoimmune diseases like systemic lupus erythematosus (SLE). However, the research on dsDNA-specific plasma cells was restricted to the ELISpot technique, with its limitations, as no other attempt for identification of dsDNA-reactive plasma cells had been successful.

**Methods:**

With improved fluorochrome labeling of dsDNA, removal of DNA aggregates, and enhanced blocking of unspecific binding, we were able to specifically detect dsDNA-reactive plasma cells by immunofluorescence microscopy.

**Results:**

Via this novel technique we were able to distinguish short-lived (SLPCs) and long-lived (LLPCs) autoreactive plasma cells, discriminate dsDNA-specific plasma cells according to their immunoglobulin class (IgG, IgM, and IgA) and investigate autoreactive (dsDNA) and vaccine-induced ovalbumin (Ova) plasma cells in parallel.

**Conclusions:**

The detection of autoreactive dsDNA-specific plasma cells via immunofluorescence microscopy allows specific studies on pathogenic and protective plasma cell subsets and their niches, detailed evaluation of therapeutic treatments and therefore offers new possibilities for basic and clinical research.

**Electronic supplementary material:**

The online version of this article (doi:10.1186/s13075-015-0811-2) contains supplementary material, which is available to authorized users.

## Introduction

Pathogenic self-reactive plasma cells (PCs) have been in the focus of autoimmunity research for many years since they contribute to the onset and maintenance of the disease [1–5]. In humans and mice alike antinuclear antibodies (ANAs) are a main feature and hallmark of autoimmunity [6]. In systemic lupus erythematosus (SLE) especially, double-stranded deoxyribonucleic acid (dsDNA)-specific autoantibodies are associated with the pathogenesis and correlate with disease activity [7]. Antibodies against dsDNA are produced by short-lived (SLPCs) and long-lived plasma cells (LLPCs). While the first are mainly associated with flares the latter are refractory against most immunosuppressive treatments and contribute to chronicity and relapses of the disease [1]. However, LLPCs are not long-lived per se but need permanent anti-apoptotic stimuli, which are supplied by the microenvironment. This so-called survival niche [2] consists of stromal (SNC) and hematopoietic niche components (HNCs) [8, 9]. Most data about LLPCs are acquired by studies on total PCs or from responses against protein model vaccines for protective PCs such as ovalbumin (Ova). In contrast, the knowledge about pathogenic dsDNA-specific PCs is rare as no identification of dsDNA-specific PCs has been possible in histology. However, detailed and comparative studies appear essential as many aspects, such as the characteristics of the antigen or the B cell stimulation/activation route, modify the PC fate and the turnover within the memory PC compartment [10–13]. Moreover, subsets of memory PCs may reside in alternative niches [14, 15], which might be differently modified by therapeutic treatments [16].

All these factors influence the protective and pathogenic humoral memory, disease progression and therapeutic success. Thus, we wanted to establish a method that allows the identification of autoreactive (dsDNA-specific) PCs and comparative studies with PCs of different antigen specificities (e.g., pathogenic (dsDNA) vs. protective (Ova) PCs) within the tissue. Moreover, the method should enable a distinction of dsDNA LLPCs and SLPCs, investigation of dsDNA PCs of different immunoglobulin (Ig) classes and the evaluation of therapeutic treatments.

## Methods

### Mouse strains and treatments

Female mice from autoimmune (NZB/W F1 and BcN/LmoJ) and non-autoimmune (C57BL/6) strains were bred at the animal facility of the Deutsches Rheuma-Forschungszentrum (DRFZ) and C57BL/6 mice with experimental autoimmune myasthenia gravis (EAMG) were kept at the animal facility of the Charité - Universitätsmedizin Berlin under defined pathogenic-free conditions and treatments were approved (approval number G130/08, G113/10 and G0172/13) by the Landesamt für Gesundheit und Soziales (LaGeSo) Berlin, Germany. All animal experiments were performed by certified personnel and all efforts were made to minimize suffering. For dsDNA analysis, mice with positive dsDNA antibody titer (between 5 and 7 months old) and age-matched controls were chosen. Experimental autoimmune myasthenia gravis was induced by three intraperitoneal injections of torpedo acetylcholine receptor (AChR) in Freund’s complete adjuvant into 3-month-old C57BL/6 mice at 4-week intervals. Mice were sacrificed for analysis 17 days after the last injection at an age of 6 months and after conformation of active EAMG by positive anti-AChR antibody titer and disease score [17]. For distinction of autoreactive LLPCs and SLPCs, 6-month-old BcN/LmoJ mice (another mouse model with genetic predisposition for SLE, which was backcrossed to the C57BL/6 strain and thus is also known as the B6.Sle123 strain [18]) with positive dsDNA antibody titer were fed 5-ethynyl-2′-deoxyuridine (EdU) (0.3 mg/ml) for 2 weeks.

### Labeling of dsDNA

Five hundred micrograms of dsDNA (activated dsDNA from calf thymus (Sigma-Aldrich Chemie GmbH, Munich, Germany)) in 500 μl phosphate-buffered saline (PBS) was reduced with 10 μl 0.5 mol/l dithiothreitol for 30 min at room temperature. Subsequently, the labeling solution was rebuffered with Amicon Ultra (10 kDa) in borate buffer (pH9.5) with 10 mg/ml DMSO containing 20 μl digoxigenine (dig-NHS, Sigma-Aldrich) and incubated for 1 h at room temperature in the dark. After labeling, reaction-free dig was removed in a PD10 desalting column equilibrated with PBS/0.05 % azide and fractions containing high concentrations of labeled dsDNA were collected and pooled.

### Sample preparation and immunofluorescence staining for histology

Spleen and bone marrow samples were embedded in O.C.T. compound medium (Sakura Alphen aan den Rijn, The Netherlands) in cryomold vessels, snap frozen in liquid nitrogen for 10 min and stored at −80 °C. Kryosections of 8 μm thickness were prepared and subsequently fixated with acetone at −20 °C for 10 min, air dried and stored at −80 °C.

For blocking, sections were rehydrated with RPMI 1640 medium (Gibco, Waltham, MA, USA) containing 10 % fetal calf serum (FCS) and anti-FcyRII/III antibody (clone 2.4G2, DRFZ). Samples for antigen-blocking control additionally contained unlabeled dsDNA (7 μl of 1 mg/ml to 70 μl). After 1 h, dsDNA-dig was centrifuged to remove DNA aggregates and immediately added to the sample. Sections were incubated overnight at 4 °C. The following day, samples were washed and incubated with anti-Ig light chain kappa (IgL)-rPE (clone 187.1, DRFZ) for plasma cell identification [19]. The secondary staining solution containing anti-dig-Cy5 (prepared and labeled at DRFZ) was applied for 30 min. Stained samples were mounted with fluoromount (Dako, Glostrup, Denmark) and analyzed by confocal laser scanning microscopy (Zeiss LSM 710, Zeiss, Oberkochen, Germany).

For parallel staining of pathogenic and protective PCs, samples were incubated with Ova-FITC (Sigma-Aldrich, labeled at DRFZ) together with secondary staining solution.

For identification of Ig-class of dsDNA PCs, sections were incubated with anti-IgA-bio (goat, SouthernBiotech, Birmingham, AL, USA), anti-IgG-FITC (rabbit, SouthernBiotech) and anti-IgM-Pacific blue (clone M41, DRFZ) instead of anti-IgL and subsequently incubated with streptavidin-rPE (BD Biosciences, Franklin Lakes, NJ, USA).

For distinction of proliferating SLPCs and non-proliferating LLPCs, incorporated EdU was detected with the click-it® EdU kit (Life Technologies, Carlsbad, CA, USA) according to the manufacturer’s instruction.

### ELISpot analysis

#### Enzymatic ELISpot

Ninety-six-well microtiter plates (Merck Millipore, Billerica, MA, USA) were precoated with methyl-bovine serum albumin (BSA) (Sigma-Aldrich) and subsequently coated with calf thymus DNA (Sigma-Aldrich) as previously described [20]. Single-cell suspensions were prepared in RPMI 1640 medium supplemented with 10 % FCS (Invitrogen, Waltham, MA, USA), penicillin, streptomycin, and glutamine (complete medium). The cells were then pipetted onto the plates and incubated at 37 °C for 3 h in a 5 % CO_2_-containing incubator. Afterward, cells were washed away and plates were incubated with biotin-labeled goat anti-mouse IgM or IgG (SouthernBiotech) followed by ExtrAvidin-Alkaline Phosphatase (Sigma-Aldrich). The spots were developed with NBT/BCIP (Thermo Fisher Scientific, Waltham, MA, USA) and enumerated by an automatic ELISpot Reader (AID Autoimmun Diagnostika, Strassberg, Germany) using the ELISpot Reader software (AID Autoimmun Diagnostika).

#### Fluorescence ELISpot

Ninety-six-well microtiter plates (Merck Millipore) were coated with goat anti-mouse IgG and IgM (SouthernBiotech) respectively or together. For fluorescence acquisition of dsDNA-specific PCs the probes were incubated with dsDNA-dig followed by anti-dig-A594 and for total PCs with anti-IgG and IgM-bio (SouthernBiotech) followed by streptavidin-FITC (BD Biosciences). ELISpot analysis were performed blinded and independently to histologic analysis.

### ELISA

Blood was drawn from the left heart ventricle directly after cervical dislocation of the mouse. Serum was prepared and analyzed as previously described [21]. Briefly, 96-well microtiter plates were precoated with methyl-BSA (Sigma-Aldrich; 1 mg/mL) at 37 °C for 3 h and subsequently coated with calf thymus DNA (Sigma-Aldrich; 1 mg/mL) at 4 °C overnight. After blocking with PBS/3 % BSA, serum was added and incubated overnight at 4 °C. The next day, the ELISA was developed by addition of biotin-labeled goat anti-mouse IgM or IgG (SouthernBiotech), ExtrAvidin-peroxidase (Sigma-Aldrich) and TMB substrate (Thermo Fisher Scientific).

### Data analysis and statics

Histology data were analyzed with Zen2012 (Zeiss), ELISpot data with ELISpot6 (AID Autoimmun Diagnostika), and graphs were prepared with Prism 5 (Graphpad Software Inc., San Diego, CA, USA). Results are expressed as means ± standard deviation (SD) and groups were compared by unpaired Student’s *t* test. Correlation between matched data points from histologic and ELISpot analysis were calculated via Pearson test. *P* values <0.05 were considered significant differences or correlation respectively and are indicated by one star (^*^), values <0.01 with two stars (^**^) and values <0.001 with three stars (^***^).

## Results and discussion

### Immunofluorescence staining of dsDNA-reactive plasma cells for histologic analysis

**Fig. 1:**
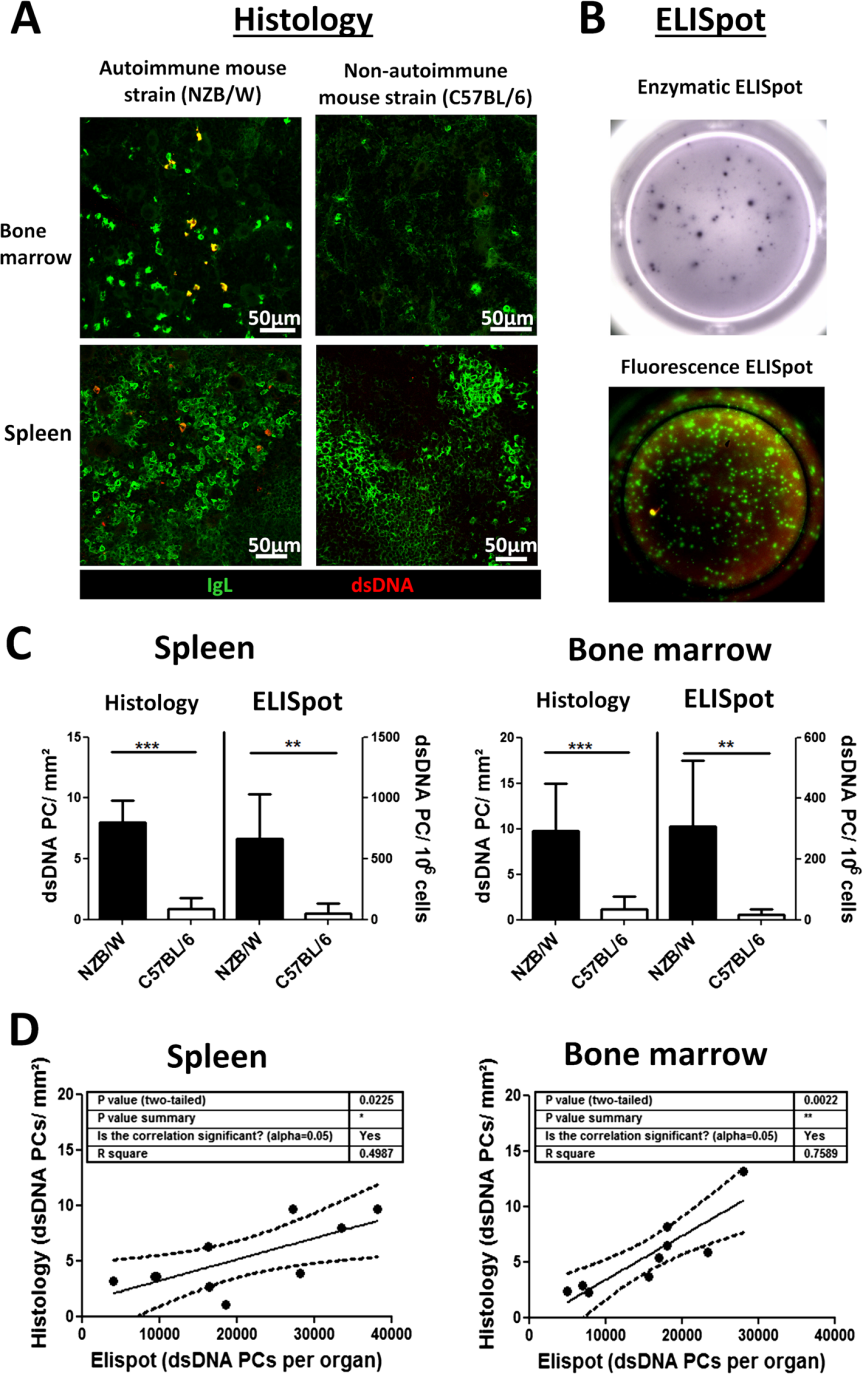
Analysis of double-stranded deoxyribonucleic acid (dsDNA)-specific plasma cells (PCs) by histology and ELISpot. **a** Kryosections from bone marrows and spleens of autoimmune (NZB/W) and non-autoimmune (C57BL/6) mice were stained with anti-immunoglobulin light chain kappa (IgL) (PCs, *green*) and fluorochrome-labeled dsDNA (*red*, - > dsDNA-specific PCs = *yellow*) and analyzed by confocal laser scanning microscopy. **b** For comparison standard enzymatic ELISpot (dsDNA of all isotypes) and immunofluorescence ELISpot (dsDNA *red*; IgG, IgA and IgM *green*) are depicted. For immunofluorescence ELISpot only 5 % of original cell numbers were seeded to enable counting of total PCs in parallel. **c** Numbers of dsDNA-specific PCs acquired by histology and ELISpot. With both methods, dsDNA PCs could be identified in autoimmune (NZB/W) but none to very few in non-autoimmune (C57BL/6) mice. **d** In probes from the same NZB/W mice, numbers of dsDNA-specific PCs were assessed by histology and ELISpot. Data in spleen and bone marrow correlated significantly

Detecting dsDNA-specific PCs via immunofluorescence microscopy has not been successful so far due to demanding technical challenges of staining with labeled dsDNA that lead to no or unspecific staining. We could finally achieve this task by establishing a protocol which combined several blocking and staining conditions (see Methods). dsDNA-reactive PCs were identified by IgL staining and binding of fluorochrome-labeled dsDNA, while PCs reactive against other antigens were singularly positive for IgL (Fig. 1a and Additional file 1: Figure S1). A strong signal from PCs producing dsDNA-binding antibodies was acquired in the SLE mouse model strain (NZB/W) while in the non-autoimmune mouse strain (C57BL/6) or mice with EAMG—a disease that is not associated with anti-dsDNA antibodies—no to few positive signals were found (Additional file 1: Figure S1). Blocking with unlabeled dsDNA suppressed-reactive PCs and incubating with secondary reagent (anti-dig-Cy5) alone returned no staining (Additional file 1: Figure S1). For comparison, the standard method for detecting dsDNA-specific PCs via enzymatic or immunofluorescence ELISpot is shown as Fig. 1b. Both methods, histology and ELISpot analysis gained comparable results (Fig. 1c) and matched data sets from NZB/W mice significantly correlated (Fig. 1d). Likewise, numbers of dsDNA-specific PCs in the bone marrow and the anti-dsDNA antibody titer in the serum did (Additional file 2: Figure S2).

Whether the few dsDNA-specific PCs found in non-autoimmune C57BL/6 and EAMG mice are false positive signals marking the threshold for sensitivity of the diverse analysis methods or are indeed dsDNA-specific PCs may be disputed. However, as the dsDNA PCs appeared prominently in old (>5 month) mice and dsDNA-reactive PCs were already described in C57BL/mice [22], we tend to the latter.

### Parallel staining of pathogenic (dsDNA) and protective (Ova) plasma cells in histology

**Fig. 2:**
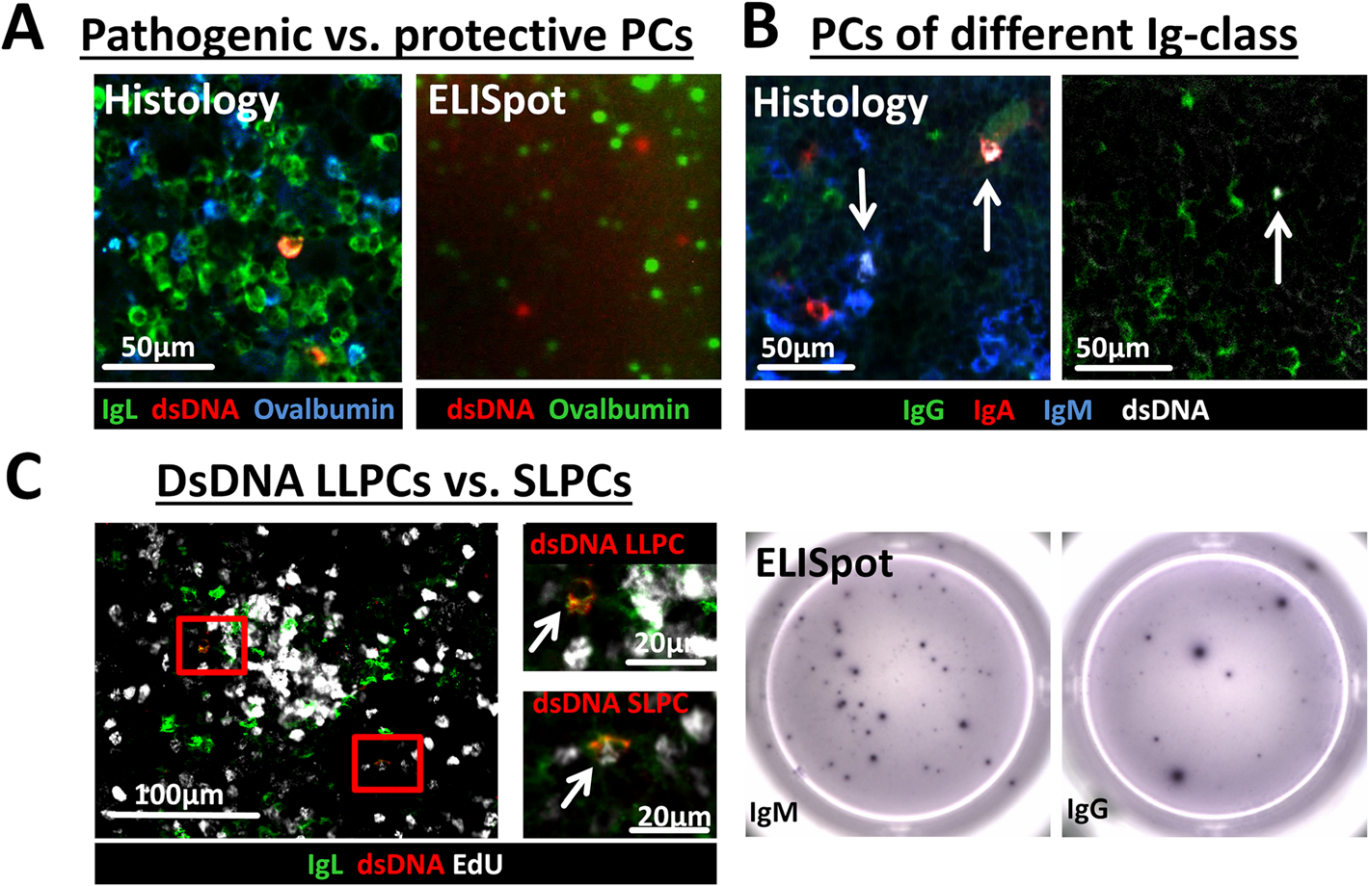
Parallel analysis of pathogenic (double-stranded deoxyribonucleic acid (dsDNA)-specific) and protective (ovalbumin (Ova)-specific) plasma cells (PCs), discrimination of dsDNA-specific PCs according to their immunoglobulin (Ig) class (IgA, IgG and IgM) and identification of autoreactive long-lived plasma cells (LLPCs) and short-lived plasma cells (SLPCs). **a** Kryosections from spleen of autoimmune mice (NZB/W) - 5 days post secondary Ova immunization - were stained with anti-Ig light chain kappa (IgL) (PCs, *green*), fluorochrome-labeled dsDNA (*red*, - > dsDNA-specific PCs = *yellow*) and fluorochrome-labeled Ova (*blue*, Ova-specific PCs = *turquoise*). PCs were either specific for dsDNA, Ova or an unknown antigen but no false double-positive dsDNA/Ova PCs occurred. For comparison, a figure of an immunofluorescence ELISpot identifying Ova (*green*) and dsDNA (*red*) antibody-secreting cells is depicted. **b** Kryosections from spleen of autoimmune (NZB/W) mice were stained with fluorochrome-labeled dsDNA (*white*), anti-IgA (*red*), anti-IgG (*green*), anti-IgM (*blue*). dsDNA-specific PCs were either IgM, IgG or IgA positive. The figures below depict dsDNA PCs of the IgM (*left*) and IgG (*right*) class acquired by ELISpot **c** Autoimmune (BcN/LmoJ) mice were fed EdU for 2 weeks. Kryosections from spleen were stained with click-it® EdU kit (EdU positive = *white*), IgL (PCs, *green*), fluorochrome-labeled dsDNA (*red*, - > dsDNA-specific PCs = *yellow*). dsDNA-specific SLPC incorporated EdU (*lower selection*, indicated by *arrow* in magnification, *white-colored nucleus*) during proliferation while dsDNA-specific LLPCs did not (*upper selection*, indicated by *arrow* in magnification, *blank nucleus*)

As nowadays treatments that deplete LLPCs [23–25] do not discriminate between protective and pathogenic PCs and erase the pathogenic and protective humoral memory alike, comparative studies between self and non-self-reactive PCs and their niches are essential [1, 26, 27]. For that purpose, we used the well-established vaccination model with ovalbumin injection, and stained dsDNA- and Ova-specific PCs in parallel in autoimmune mice. Five days after secondary Ova immunization, Ova- and dsDNA-specific PCs could be detected among PCs of unknown antigen specificity (Fig. 2a and Additional file 3: Figure S3a) and no false double-positive PCs were found.

### Distinction of dsDNA-reactive plasma cells according to their Ig class (IgG, IgA, IgM)

The Ig class of autoantibodies has impact on the pathogenesis of the disease [28] and PCs of different Ig class or antigen specificity respond differently to therapeutic treatments, which seems rather due to a manipulation of their specific niches than a direct effect on the PCs [14]. Thus, future investigations of autoreactive PCs discriminated by their Ig class seem important. We therefore counterstained dsDNA-reactive PCs with antibodies against the Ig heavy chain subclasses (IgG, IgM, and IgA) (Fig. 2b and Additional file 3: Figure S3B). No false double-positive Ig-class labeling appeared and in accordance with previous reports [21] our preliminary data indicate that the majority of dsDNA PCs were of IgM and IgG class while Ova PCs resulting from secondary systemic immunization were mainly of IgG class (data not shown) [29].

### Distinction between long-lived and newly generated short-lived autoreactive plasma cells

Due to the ubiquitous presence of autoantigens self-reactive PCs are continuously generated. As such, both long-lived dsDNA-reactive PCs in their survival niches and newly generated ones still migrating and competing for a niche are found at all times but varying in proportion. So far, three methods are possible for the investigation of LLPCs in their niche. First, via the tracking of antigen-specific PCs a long time after antigen challenge (>21 days post boost with e.g., Ova, NP, KLH or CGG) [19, 30], which is not possible for autoantigens; second, via the distinction of LLPCs and newly formed PCs due to the incorporation of bromodeoxyuridine (BrdU) or for histologic analysis more adequate EdU into the DNA of proliferating cells [31]; and third, via the staining for cell cycle proteins such as Ki67 [32]. While the latter two methods allow distinction of LLPCs and SLPCs, only the ELISpot method enables the identification of dsDNA-specific PCs. Therefore, several methods are sometimes used in parallel e.g., dsDNA ELISpot, BrdU+/- FACS and histology [7, 21] though still lacking the specific analysis of dsDNA-reactive LLPCs and their environment.

To enable the distinction between dsDNA-specific SLPCs and LLPCs, we combined EdU incorporation with staining for antigen specificity (Fig. 2c). While a proportion of dsDNA-specific and other PCs incorporated EdU indicating their recent generation, LLPCs remained EdU negative. To confirm the cytoplasmatic staining of dsDNA and the nuclear localization of EdU we added a nucleus staining with Sytox Green (Additional file 3: Figure S3C).

## Conclusions

Despite their role for autoimmunity, the research on autoreactive (dsDNA-specific) plasma cells and their survival niches was hampered by the limitations of the ELISpot technique as the only available method for the analysis of dsDNA-specific plasma cells.

The microscopic/histologic detection method described by us identifies dsDNA-specific plasma cells in equivalent numbers to the ELISpot technique but furthermore allows parallel analysis of short- and long-lived dsDNA-specific plasma cells, comparative characterization of the niches for pathogenic (dsDNA) and protective (Ova) plasma cells, and moreover a detailed evaluation of therapeutic treatments regarding changes of particular plasma cell subsets or niche components.

Therefore, this analysis method can help to reveal and understand differences between autoimmune and non-autoimmune humoral memory and may help to identify specific targets in the niche for pathogenic plasma cells.

## Additional files

Additional file 1: Figure S1. Single fluorescence channels of the dsDNA staining in tissues from autoimmune and non-autoimmune mice. (JPEG 6667 kb) Additional file 2: Figure S2. Correlation between dsDNA-specific plasma cells numbers in spleen and bone marrow with the anti-dsDNA antibody titer in the serum. (JPEG 1553 kb) Additional file 3: Figure S3. Single fluorescence channels of the combined dsDNA stainings (+Ova and + IgG, +IgA, +IgM and + EdU respectively). (JPEG 5593 kb)

## Abbreviations

AChR: acetylcholine receptor
ANA: antinuclear antibodies
BSA: bovine serum albumin
dig: digoxigenine
dsDNA: double-stranded deoxyribonucleic acid
EAMG: experimental autoimmune myasthenia gravis
EdU: 5-ethynyl-2′-deoxyuridine
FCS: fetal calf serum
HNC: hematopoietic niche component
IgL: immunoglobulin light chain kappa
LLPC: long-lived plasma cell
Ova: ovalbumin
PBS: phosphate-buffered saline
PC: plasma cell
SLE: systemic lupus erythematosus
SLPC: short-lived plasma cell
SNC: stromal niche component
