# Supplementary figures and images for: Analyzing pathogenic (double-stranded (ds) DNA-specific) plasma cells via immunofluorescence microscopy
Source: Arthritis Res Ther. 2015 Oct 21;17:293. doi: 10.1186/s13075-015-0811-2 (PMC4618946; doi:10.1186/s13075-015-0811-2)

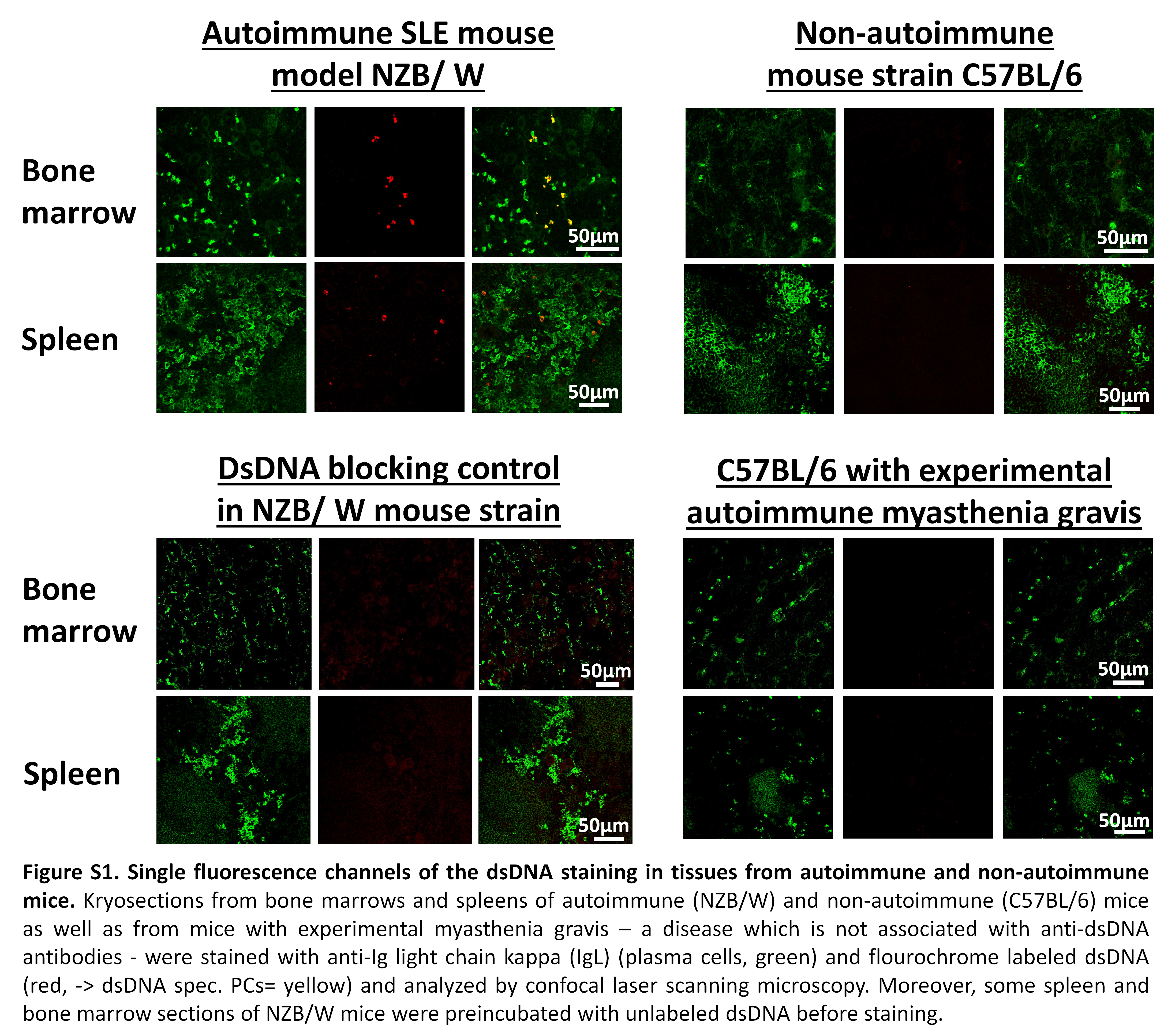

Supplement: Additional file 1: Figure S1. — Single fluorescence channels of the dsDNA staining in tissues from autoimmune and non-autoimmune mice. (JPEG 6667 kb) [file 13075_2015_811_MOESM1_ESM.jpeg]

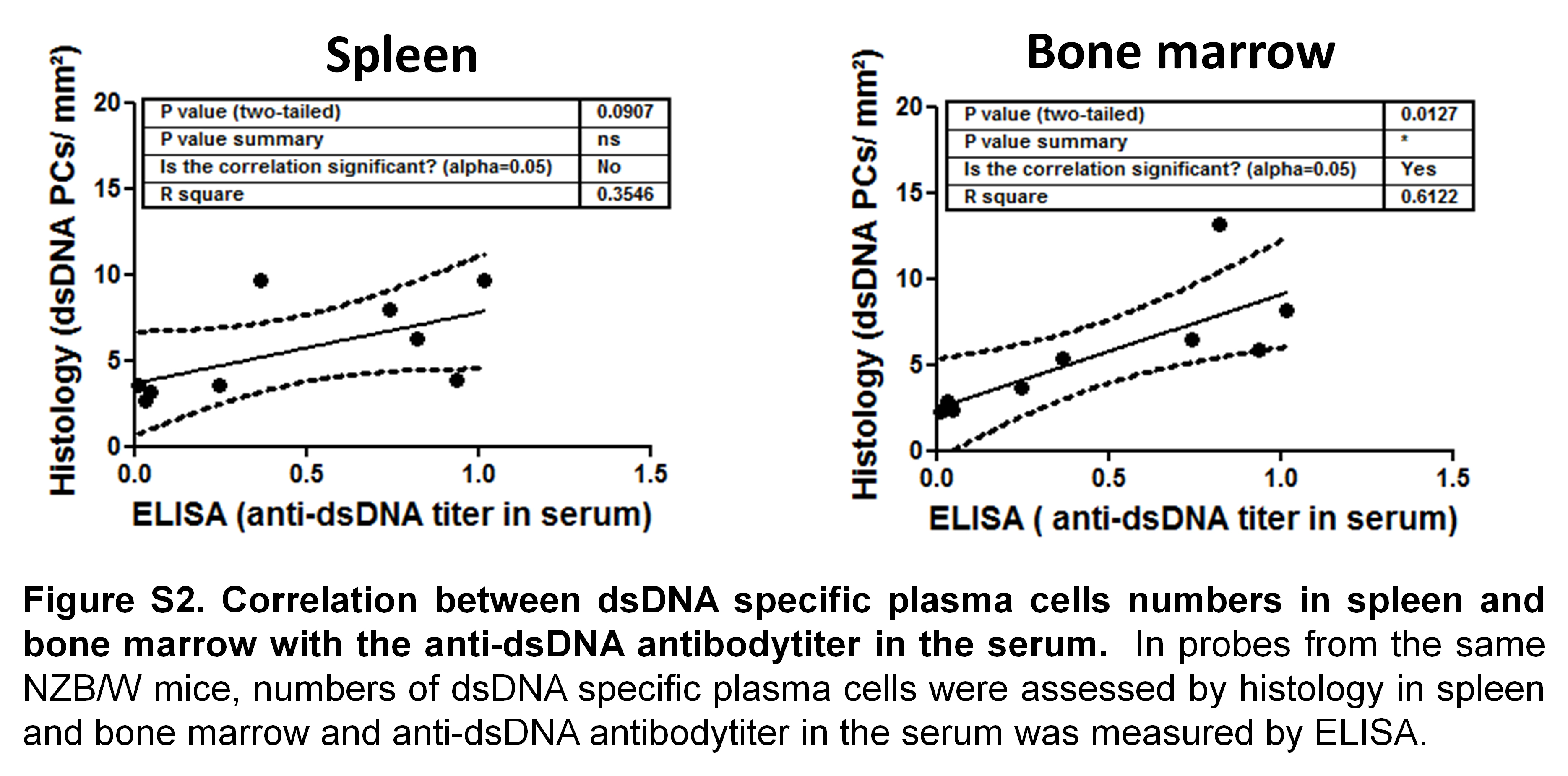

Supplement: Additional file 2: Figure S2. — Correlation between dsDNA-specific plasma cells numbers in spleen and bone marrow with the anti-dsDNA antibody titer in the serum. (JPEG 1553 kb) [file 13075_2015_811_MOESM2_ESM.jpeg]

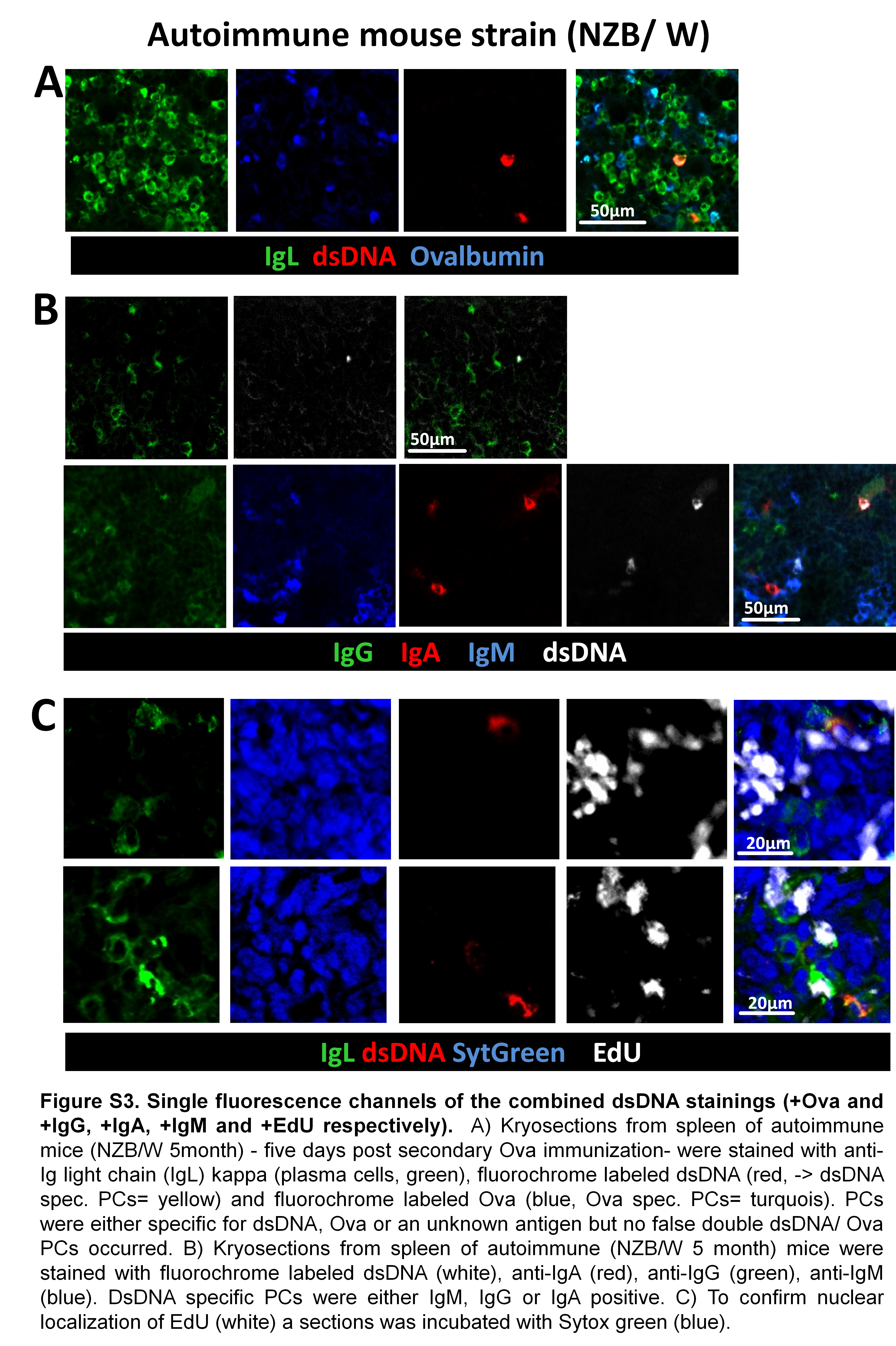

Supplement: Additional file 3: Figure S3. — Single fluorescence channels of the combined dsDNA stainings (+Ova and + IgG, +IgA, +IgM and + EdU respectively). (JPEG 5593 kb) [file 13075_2015_811_MOESM3_ESM.jpeg]
